# Maternal Probiotic or Synbiotic Supplementation Modulates Jejunal and Colonic Antioxidant Capacity, Mitochondrial Function, and Microbial Abundance in Bama Mini-piglets

**DOI:** 10.1155/2021/6618874

**Published:** 2021-05-04

**Authors:** Kai Wang, Xiangfeng Kong, Md. Abul Kalam Azad, Qian Zhu, Liang Xiong, Yuzhong Zheng, Zhangli Hu, Yulong Yin, Qinghua He

**Affiliations:** ^1^Department of Food Science and Engineering, College of Chemistry and Environmental Engineering, Shenzhen University, Shenzhen 518000, China; ^2^CAS Key Laboratory of Agro-Ecological Processes in Subtropical Regions, Hunan Provincial Key Laboratory of Animal Nutritional Physiology and Metabolic Process, National Engineering Laboratory for Pollution Control and Waste Utilization in Livestock and Poultry Production, Institute of Subtropical Agriculture, Chinese Academy of Sciences, Changsha 410125, China; ^3^Key Laboratory of Optoelectronic Devices and Systems of Ministry of Education and Guangdong Province, College of Optoelectronic Engineering, Shenzhen University, Shenzhen 518000, China; ^4^School of Food Engineering and Biotechnology, Hanshan Normal University, Chaozhou 521041, China

## Abstract

The present study was conducted to investigate the effects of maternal probiotic or synbiotic supplementation during gestation and lactation on antioxidant capacity, mitochondrial function, and intestinal microbiota abundance in offspring weaned piglets. A total of 64 pregnant Bama mini-sows were randomly allocated into the control group (basal diet), antibiotic group (basal diet + 50 g/t virginiamycin), probiotic group (basal diet + 200 mL/d probiotics per pig), or synbiotic group (basal diet + 200 mL/d probiotics per pig + 500 g/t xylo-oligosaccharides). On day 30 of post-weaning, eight piglets per group with average body weight were selected for sample collection. The results showed that maternal probiotic supplementation increased the catalase (CAT) activity in plasma and glutathione peroxidase (GSH-Px) and superoxide dismutase (SOD) activities in plasma, jejunum, and colon of piglets while decreased the malondialdehyde (MDA) and H_2_O_2_ concentrations in plasma compared with the control group (*P* < 0.05). Moreover, maternal synbiotic supplementation increased the plasma CAT activity, jejunal glutathione and GSH-Px activities, jejunal and colonic total antioxidant capacity activity, and plasma and colonic SOD activity while decreased the colonic MDA concentration of offspring piglets compared with the control group (*P* < 0.05). The mRNA levels of antioxidant enzyme-related genes (copper- and zinc-containing superoxide dismutase, nuclear factor erythroid 2-related factor 1, and nuclear factor erythroid 2-related factor 2) and mitochondrial-related genes (adenosine triphosphate synthase alpha subunit, adenosine triphosphate synthase *β*, and mitochondrial transcription factor A) in the jejunal mucosa were significantly upregulated, while the level of colonic peroxisome proliferator-activated receptor *γ* coactivator-1*α* was downregulated by maternal synbiotic supplementation (*P* < 0.05). Maternal probiotic supplementation increased (*P* < 0.05) the *Bacteroidetes* abundance in the jejunum and *Bifidobacterium* abundance in the jejunum and colon, and synbiotic supplementation increased (*P* < 0.05) the abundances of Firmicutes, *Bacteroidetes*, *Bifidobacterium*, and *Lactobacillus* in the jejunum of piglets. Furthermore, correlation analysis revealed that intestinal microbiota abundances were significantly correlated with antioxidant enzyme activities and mitochondrial-related indexes. These findings indicated that maternal probiotic or synbiotic supplementation might be a promising strategy to improve the antioxidant capacity and mitochondrial function of offspring weaned piglets by altering the intestinal microbiota.

## 1. Introduction

The gastrointestinal tract (GIT) of mammalian animals has been known as a harbor of microbes [1]. The gut microbes play crucial roles in nutrients metabolism, intestinal barrier function, and immune function [2, 3]. Accumulating evidence showed that healthy maternal gut microbes are essential for the growth and health of their offspring. Microbes can be transmitted to the offspring through direct contact with the birth canal during parturition and colostrum or milk during lactation, which contributes to long-term health benefits in offspring [4–6]. However, maternal gut microbes and milk or colostrum quality are influenced by maternal diet compositions, and thus, maternal dietary intervention may be an effective way to improve offspring's overall health.

Piglets are highly susceptible to intestinal structural abnormalities and functional disorders due to their immature immune system and lack of diverse intestinal microbiota, resulting in increased incidence of diarrhea, growth retardation, and even death. Oxidative stress occurs under a condition when the production of reactive oxygen (ROS) and their elimination by the antioxidant mechanism is imbalanced. The sows undergo systematic oxidative stress during late pregnancy and lactation, which does not fully recover until weaned and could affect their offspring's health [7]. Furthermore, there is also a high correlation between maternal and fetal (cord blood) plasma antioxidant markers, suggesting that maternal oxidative stress status can transfer to the fetus [8]. Therefore, late gestation and early postnatal periods are the critical window periods for oxidative stress regulation [9].

Probiotics are defined as “live microorganisms which when administered in adequate amounts confer a health benefit on the host” [10]; prebiotics are defined as a “nonviable food component that confers a health benefit on the host associated with modulation of the microbiota”; Gibson and Roberfroid introduced the term “synbiotic” to describe a combination of synergistically acting probiotics and prebiotics [11, 12]. Currently, increasing evidence has indicated that probiotic/synbiotic supplementation could improve antioxidant capacity and reduce oxidative stress [13]. *Lactobacillus acidophilus* and *Lactobacillus bulgaricus*, as the lactic acid bacteria, have been reported to exhibit antioxidant capacity by chelating metal ions and scavenging ROS [14]. Nie et al. reported that *Lactobacillus frumenti* improved antioxidant capacity via nitric oxide production mediated by nitric oxide synthase 1 activation in intestinal epithelial cells [15]. As a probiotic yeast, *Saccharomyces cerevisiae* (*S. cerevisiae*) showed strong antioxidant activity, reducing nitric oxide and hydroxyl radical scavenging activity [16]. Several studies have reported that the administration of probiotics or synbiotics during gestation and lactation have been considered as a potential strategy to improve the growth performance and modulate intestinal microbiota of offspring piglets [17–19]. Furthermore, maternal dietary fiber supplementation during gestation has an important role in improving the antioxidative capacity of their offspring through modulating the composition of the gut microbiota [20]. However, whether maternal probiotic or synbiotic supplementation during gestation and lactation can change the intestinal antioxidant capacity and mitochondrial function of offspring by altering the gut microbiota remains unclear.

Our previous study has found that dietary synbiotic supplementation to sows during pregnancy and lactation can improve piglet's survival and lipid metabolism by altering gut microbiota diversity and composition [21]. Therefore, we hypothesized that maternal probiotic or synbiotic supplementation could improve the antioxidant capacity and mitochondrial function by altering the gut microbiota of offspring weaned piglets. Considering that Bama mini–pigs' anatomy and physiology are similar to humans' [22, 23], Bama mini–pigs were chosen as a research model in this study. Our findings may, in turn, have important implications for understanding the link between maternal diets and infant intestinal health.

## 2. Materials and Methods

### 2.1. Experimental Design and Animal Management

The animal use and animal trials in this study have been approved by the Animal Care and Use Committee of the Institute of Subtropical Agriculture, Chinese Academy of Sciences.

A total of 64 pregnant Bama mini–sows with similar physical conditions with 3-5 parities were randomly assigned to the control group (sows fed a basal diet), antibiotic group (sows fed a basal diet supplemented with 50 g/t virginiamycin), probiotic group (sows fed a basal diet supplemented with 200 mL/d probiotic fermentation broth per pig), or synbiotic group (sows fed a basal diet supplemented with 200 mL/d probiotic fermentation broth per pig and 500 g/t xylo-oligosaccharides). From mating (day 0) to day 104 of pregnancy, the sows were individually housed in gestation crates (2.2 m × 0.6 m). On day 105, the sows were transferred to individual farrowing units (2.2 m × 1.8 m).

After weaning on day 28, two piglets per litter close to the average body weight were selected for the remaining of the trial, and four piglets from the same group were fed in one pen, and each group consisted of eight pens (replicates). Basal diets for the sows and piglets designed according to (NY/T65-2004), the Chinese nutrient requirements of swine in china (Supplementary Table 1 and Table 2) [24]. Sows were fed twice daily (at 8 : 00 and 17 : 00) according to their body conditions. Sows and piglets had available *ad libitum* access to water during the trial period. Hunan Lifeng Biotechnology Co., Ltd. (Changsha, China) provided the viable probiotic fermentation broth (*Lactobacillus* *plantarum* B90 (CGMCC1.12934) ≥ 1.0 × 10^8^ CFU/g; *S*.*cerevisiae* P11 (CGMCC2.3854) ≥ 0.2 × 10^8^ CFU/g). Shandong Longlive Biotechnology Co., Ltd. (Dezhou, China) afforded the xylo-oligosaccharides (XOS), which contained the xylobiose, xylotriose, and xylotetraose (≥35%).

### 2.2. Sampling

On day 30 of post–weaning, eight piglets (one piglet per replicate) from each group were selected and weighed at 12 h after the last feeding. Blood samples (10 mL) were collected from the precaval vein into heparin-treated tubes, and the plasma was obtained by centrifuging at 3,500 g for 10 min at 4°C and then stored at −20°C for further analysis. The piglets were then sacrificed using electrical stunning (120 V, 200 Hz), and the contents of the jejunum (10 cm below the flexure of duodenum-jejunum) and colon (middle position) were collected, immediately frozen in liquid nitrogen, and stored at −80°C for bacterial DNA extraction. In addition, the intestinal tissues of the jejunum and the colon were excised and rinsed with ice-cold physiological saline. The mucosa scrapings were collected, immediately frozen in liquid nitrogen, and stored at −80°C for further analyses.

### 2.3. Determination of Plasma and Intestinal Mucosa Antioxidant Capacity

Approximately 100 mg of frozen jejunum and colon tissues was removed quickly and homogenized with ice-cold physiologic saline (1 : 9, *w*/*v*) and then centrifuged at 2,000 g for 20 min at 4°C. The intestinal supernatants were used for further analysis. Plasma and intestinal antioxidant indicators, including catalase (CAT), superoxide dismutase (SOD), glutathione (GSH), and glutathione peroxidase (GSH-Px), as well as malondialdehyde (MDA) were analyzed by ELISA assay kits from Jiangsu Meimian Institute (Mei mian, Yancheng, China). The total antioxidant capacity (T-AOC) and H_2_O_2_ assay kits were purchased from Nanjing Jiancheng Bioengineering Institute (Nanjing, China). The test for each index was carried out according to the instructions of the kits. The absorbance values were read on a Multiscan Spectrum Spectrophotometer (Tecan, Infinite M200 Pro, Switzerland). The jejunal and colonic mucous antioxidant parameters were normalized to the total protein concentration (mg/L) quantified by the Pierce BCA Protein Assay Kit (CoWin Biosciences, Suzhou, China).

### 2.4. Determination of Intestinal Mucosa ATP Concentration

The ATP concentration of the jejunal and colonic mucosa was determined using the ATP assay kit (Mei mian, Yancheng, China) based on firefly luciferase by a Multiscan Spectrum Spectrophotometer (Tecan, Infinite M200 Pro, Switzerland). The methods for intestinal tissue homogenization and total protein quantification were the same as mentioned above.

### 2.5. Determination of Mitochondrial Complex I and III Activities

The NADH ubiquinone oxidoreductase complex I and III activities in the jejunal and colonic mucosa were assessed using commercially available kits (Comin bio. Co., Suzhou, China), according to the manufacturer's instructions. The methods for intestinal tissue homogenization and total protein quantification were the same as mentioned above. The complex I and III activities were normalized to the total protein.

### 2.6. Real-Time Quantitative PCR Analysis of Intestinal Mucosa Antioxidant-Related Genes and Mitochondrial-Related Genes

Total RNA was extracted from the frozen jejunal and colonic mucosa using a Trizol Reagent (Magen, Guangzhou, China) according to the manufacturers' protocol. The total RNA (1,000 ng) was used as a template for the cDNA reaction, which was synthesized using a PrimeScript RT reagent kit with gDNA Eraser (TaKaRa, Dalian, China). Real-time PCR analysis was performed on the LightCycler® 480 II Real-Time PCR System (Roche, Basel, Swiss) (384-cell standard block). Pig-specific primers were designed and synthesized by Sangon Biotech (Shanghai, China) Co., Ltd (Table 1). The specificity of the primers was examined by the Primer-BLAST tool (https://www.ncbi.nlm.nih.gov/tools/primer-blast) and confirmed by single peaks in the melting curves. The reaction mixture (10 *μ*L) consisted of 5.0 *μ*L SYBR Premix Ex TaqTM (AG11701; Accurate Biotechnology, Changsha, China), 2.0 *μ*L of template DNA, 0.25 *μ*L of each primer, and 2.5 *μ*L of double-distilled water. The PCR amplification conditions were followed according to the instructions of SYBR Green Premix. The relative levels of the gene expression were analyzed using 2^-*ΔΔ*Ct^ value, and the reference gene *β*-actin was used as an internal control.

### 2.7. Quantification of the Intestinal Mucosa Mitochondrial DNA Content

Total DNA was extracted from the jejunal and colonic mucosa of each piglet using a DNAiso Reagent according to the manufacturer's protocol (Accurate Biotechnology, Changsha, China). The concentration of extracted DNA was measured at 260 nm with a NanoDrop One Microvolume UV-Vis Spectrophotometer (Thermo Scientific, Waltham, USA), and the extracted DNA was stored at −20°C until further use. The content of mitochondrial DNA (mtDNA) relative to the nuclear genomic DNA was measured by amplifying the mt D-loop and nuclear-encoded *β*-actin gene using a real-time PCR assay as described above. The primer sequences for mt D-loop and G6PC are presented in Table 2. The mtDNA expression 2^-*ΔΔ*Ct^ value was calculated, and the reference gene G6PC was used as an internal control.

### 2.8. Real-Time Quantitative PCR Analysis for Jejunal and Colonic Microbiota Abundances

The total bacterial genomic DNA was extracted from 300 mg of jejunal and colonic luminal contents using a Mag-Bind® Stool DNA Kit (Omega, Guangzhou, China) according to the manufacturer's protocol. The primers of the selected genes are listed in Table 3. The reaction mixture (10 *μ*L) consisted of 5.0 *μ*L SYBR Premix Ex Taq TM (AG11701; Accurate Biotechnology, Changsha, China), 2.0 *μ*L of template DNA, 0.25 *μ*L of each primer, and 2.5 *μ*L of double-distilled water. The standard curves of each gene were generated with 10-fold serial dilutions of the respective 16S rRNA genes [25]. The qPCR amplification was carried out according to the instructions of SYBR green premix (Takara Biotechnology, Dalian, China). Melting curves were checked for each gene after amplification. The results are expressed as Lg16S ribosomal DNA gene copies/g intestine contents.

### 2.9. Statistical Analysis

Statistical analyses were performed using the SPSS 22.0 statistical software (SPSS Inc., Chicago, IL). Data were analyzed by one-way ANOVA, and comparative analyses were conducted using the Tukey post hoc test. Statistical results are presented as means ± standard error of the mean (SEM). Differences were considered significant if *P* < 0.05, and 0.05 ≤ *P* < 0.10 was considered a trend. The R package of “Hmisc” was used for calculating Spearman's correlation coefficient.

## 3. Results

### 3.1. Effect of Maternal Probiotic or Synbiotic Supplementation on Piglets' Plasma Redox Status

**Figure 1:**
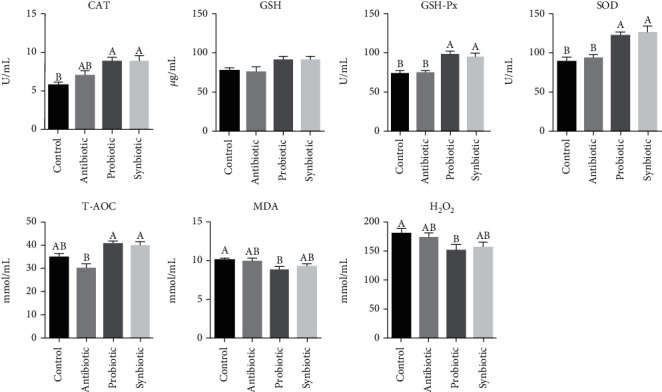
Effect of maternal probiotic or synbiotic supplementation during gestation and lactation on piglets' plasma redox status. Data are expressed as means ± SEM (*n* = 8). Values with different letters mean statistically significant differences (*P* < 0.05). Values with no letters mean no statistically significant differences among the groups (*P* > 0.05). CAT: catalase; GSH: glutathione; GSH-Px: glutathione peroxidase; SOD: superoxide dismutase; T-AOC: total antioxidant capacity; MDA: malondialdehyde.

To explore whether maternal probiotic or synbiotic supplementation contributes to the systemic redox status, we determined the antioxidant and oxidative parameters in plasma. As shown in Figure 1, in comparison with the control group, the activities of CAT, GSH-Px, and SOD were higher, whereas the concentrations of MDA and H_2_O_2_ were lower in the probiotic group (*P* < 0.05). Synbiotic supplementation increased (*P* < 0.05) plasma CAT, GSH-Px, and SOD activities compared with the control group. Meanwhile, plasma GSH-Px, SOD, and T-AOC activities in the probiotic and synbiotic groups were elevated (*P* < 0.05) compared with the antibiotic group. These findings suggest that maternal probiotic or synbiotic supplementation could improve the systemic antioxidant capacity of offspring piglets.

### 3.2. Effect of Maternal Probiotic or Synbiotic Supplementation on Piglets' Intestinal Antioxidant Capacity

**Figure 2:**
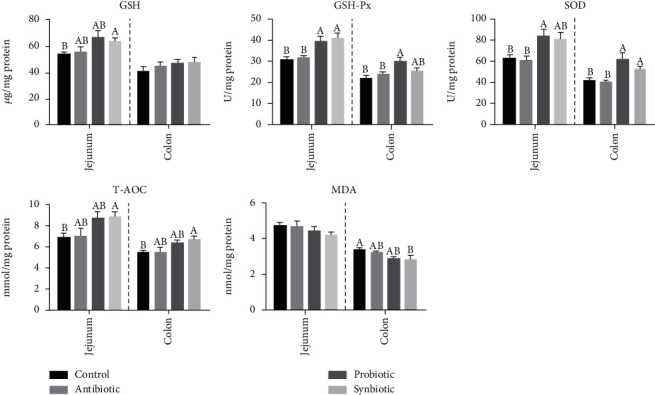
Effect of maternal probiotic or synbiotic supplementation during gestation and lactation on piglets' oxidant/antioxidant status in the jejunum and colon. Data are expressed as means ± SEM (*n* = 8). Values with different letters mean statistically significant differences (*P* < 0.05). Values with no letters mean no statistically significant differences among the groups (*P* > 0.05). GSH: glutathione; GSH-Px: glutathione peroxidase; SOD: superoxide dismutase; T-AOC: total antioxidant capacity; MDA: malondialdehyde.

We further investigated the role of probiotic or synbiotic on jejunal and colonic antioxidant capacity. As shown in Figure 2, in the jejunum, the activities of GSH-Px and SOD were increased in the probiotic group (*P* < 0.05), and the GSH, GSH-Px, and T-AOC activities were increased in the synbiotic group (*P* < 0.05) when compared with the control group. In addition, maternal probiotic supplementation increased (*P* < 0.05) the GSH-Px and SOD activities, while maternal synbiotic supplementation increased (*P* < 0.05) the GSH-Px activity compared with the antibiotic group. In the colon, compared with the control group, the GSH-Px and SOD activities were higher (*P* < 0.05) in the probiotic group, as well as the SOD and T-AOC activities in the synbiotic group, while the MDA concentration was lower (*P* < 0.05) in the synbiotic group. In addition, the GSH-Px and SOD activities in the probiotic group and the SOD activity in the synbiotic group were increased (*P* < 0.05) in comparison to the antibiotic group. Therefore, these results indicate that maternal probiotic or synbiotic supplementation facilitates improving the intestinal antioxidant capacity in offspring piglets.

### 3.3. Effect of Maternal Probiotic or Synbiotic Supplementation on Piglets' Intestinal Antioxidant Enzyme-Related Genes

To investigate the molecular mechanism by which maternal probiotic or synbiotic supplementation may influence the intestinal antioxidant function, the expression of genes related to the antioxidant capacity was determined. Maternal synbiotic supplementation upregulated (*P* < 0.05) the relative mRNA expressions of CuZnSOD, Nrf1, and Nrf2 in the jejunum compared with the control and antibiotic groups. The mRNA expression of GPx1 was tended to upregulate in the probiotic (*P* = 0.090) and synbiotic (*P* = 0.068) groups compared with the control group. Interestingly, the mRNA expressions of GPx4, Nrf1, and Nrf2 in the colon were lower (*P* < 0.05) in the antibiotic group than those in the control group. Meanwhile, the mRNA expression of colonic Nrf2 in the probiotic (*P* = 0.095) and synbiotic (*P* = 0.087) groups was tended to increase compared with the antibiotic group (Table 4). Our data indicated that maternal probiotic or synbiotic supplementation could improve the jejunal antioxidant enzyme-related genes to some extent, while had no significant effect on the colon.

### 3.4. Effect of Maternal Probiotic or Synbiotic Supplementation on Piglets' Intestinal ATP Concentrations, Mitochondrial Complex I and III Activities

As enterocytes have high energy demands to maintain renewal and the transport of nutrients, the effect of maternal probiotic or synbiotic supplementation on the mitochondrial oxidative metabolism and the jejunal and colonic mucosal ATP production were measured. Although there were no significant differences in the jejunal ATP concentration among the four groups, the concentration of jejunal ATP was higher by 9.1% and 18.7% in the probiotic and synbiotic groups, respectively, than the control group (Figure S1). The jejunal mitochondrial complex I activity in the antibiotic group was higher compared with the other three groups. Compared to the control group, the activity of colonic mitochondrial complex I was higher by 20.16% and 44.82% in the antibiotic and probiotic groups, while tended to decreased (*P* = 0.086) in the synbiotic group (Figure S2A). However, there were no significant differences in the mitochondrial complex III activity in the jejunum and colon among the four groups (Figure S2B). These findings suggest that maternal probiotic or synbiotic supplementation tends to improve jejunal mitochondrial function.

### 3.5. Effect of Maternal Probiotic or Synbiotic Supplementation on Piglets' Mitochondrial Biogenesis-Related Genes in the Jejunum and Colon

Subsequently, mitochondrial biogenesis-related genes in the intestinal mucosa were measured to understand further protective effects of maternal probiotic or synbiotic on mitochondria. As shown in Table 5, in the jejunal mucosa, maternal synbiotic supplementation increased (*P* < 0.05) the mRNA expressions of ATP5A1, ATP5B, and TFAM compared to the control group, as well as the mRNA expressions of ATP5A1 and ATP5B compared with the antibiotic group. In comparison with the probiotic group, maternal synbiotic supplementation increased (*P* < 0.05) the mRNA expression of ATP5B in piglets' jejunum. However, in the colonic mucosa, the PGC1*α* mRNA expression level was decreased (*P* < 0.05) in the synbiotic group compared with the control group. The results demonstrated that maternal synbiotic supplementation could improve the jejunal mitochondrial biogenesis-related genes.

### 3.6. Effect of Maternal Probiotic or Synbiotic Supplementation on Piglets' Microbial Abundances in the Jejunum and Colon

**Figure 3:**
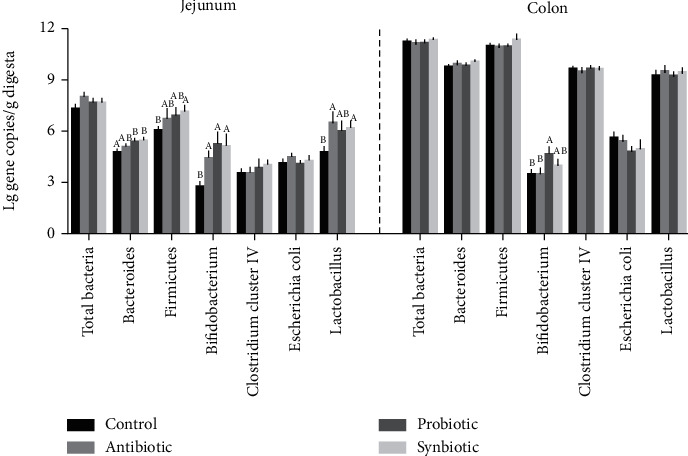
Effect of maternal probiotic or synbiotic supplementation during gestation and lactation on the copy numbers (Lg (copies/g)) of bacterial abundance in offspring piglets. Data are expressed as means ± SEM (*n* = 8). Values with different letters mean statistically significant differences (*P* < 0.05). Values with no letters mean no statistically significant differences among the groups (*P* > 0.05).

We further investigated the effects of maternal probiotic or synbiotic supplementation on the selected intestinal microbiota abundances in offspring piglets. As shown in Figure 3, in the jejunum, maternal probiotic supplementation increased (*P* < 0.05) the relative abundances of *Bacteroidetes* and *Bifidobacterium*, and synbiotic supplementation increased (*P* < 0.05) the relative abundances of *Firmicutes*, *Bacteroidetes*, *Bifidobacterium*, and *Lactobacillus* when compared with the control group. Similarly, maternal antibiotic supplementation also increased the relative abundances of total bacteria (*P* = 0.066), *Bifidobacterium* (*P* < 0.05), and *Lactobacillus* (*P* < 0.05) compared with the control group. In the colon, the relative abundance of *Bifidobacterium* in the probiotic group was higher (*P* < 0.05) compared with the control and antibiotic groups. However, there were no significant differences in the abundances of total bacteria, *Bacteroides*, *Clostridium cluster* IV, and *Lactobacillus* among the four groups. These results indicated that maternal probiotic or synbiotic significantly changed particular bacteria abundances in offspring piglets, with an increase in the counts of beneficial bacteria in the jejunum.

### 3.7. Correlation Analysis of Antioxidant Index, Mitochondrial Index, and Intestinal Bacteria Abundance in the Jejunum and Colon

**Figure 4:**
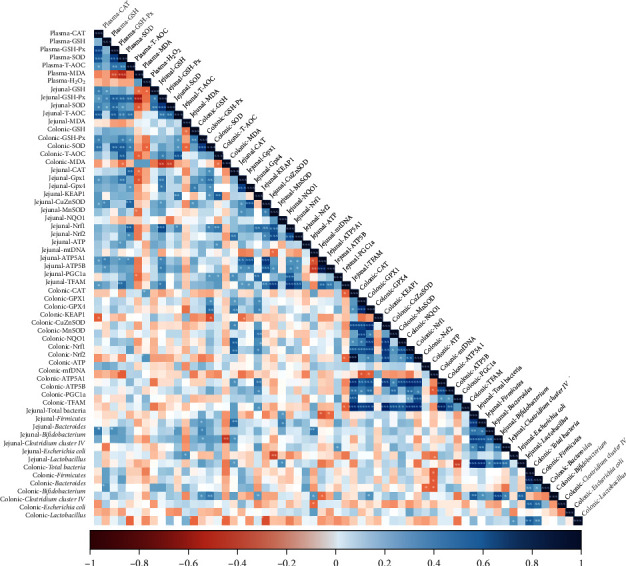
Correlation of antioxidant indexes, mitochondrial function indexes, and bacteria abundances. The R package of “corroplot” was used for generating the heat maps. The blue color represents a significant positive correlation, and red color represents a significant negative correlation. Asterisks indicate statistically significant difference: ^∗^*P* < 0.05; ^∗∗^*P* < 0.01; ^∗∗∗^*P* < 0.001. CAT: catalase; GSH: glutathione; GSH-Px: glutathione peroxidase; SOD: superoxide dismutase; T-AOC: total antioxidant capacity; MDA: malondialdehyde; GPx1: glutathione peroxidase 1; GPx4: glutathione peroxidase 4; Keap1: kelch-like ECH-associated protein 1; CuZnSOD: copper- and zinc-containing superoxide dismutase; MnSOD: manganese-containing superoxide dismutase; NQO1: nicotinamide adenine dinucleotide (phosphate) dependency quinone oxidoreductase 1; Nrf1: nuclear factor erythroid 2-related factor 1; Nrf2: nuclear factor erythroid 2-related factor 2; mtDNA: mitochondrial DNA; ATP5A1: adenosine triphosphate synthase alpha subunit; ATP5B: adenosine triphosphate synthase *β*, polypeptide; PGC-1*α*: peroxisome proliferator-activated receptor *γ* coactivator-1*α*; TFAM: mitochondrial transcription factor A.

The correlation analysis of all measured bacterial abundance, antioxidant index, and mitochondrial function parameters is shown in Figure 4. The correlation analysis revealed that jejunal *Bifidobacterium* abundance was positively correlated (*P* < 0.05) with the jejunal CAT expression and colonic GSH-Px activity but negatively correlated (*P* < 0.05) with the colonic MDA concentration. The jejunal *Escherichia coli* abundance was positively correlated (*P* < 0.05) with jejunal MDA concentration, while negatively correlated (*P* < 0.05) with the jejunal MnSOD expression and colonic mtDNA expression. The jejunal *Lactobacillus* abundance was positively correlated (*P* < 0.05) with the jejunal CAT and NQO1 expression. The jejunal *Bacteroidetes* was positively correlated (*P* < 0.05) with plasma CAT and GSH-Px activities, jejunal T-AOC activity and mtDNA expression, and colonic T-AOC activity. The jejunal Firmicutes abundance was positively correlated (*P* < 0.05) with colonic GSH-Px activity and jejunal CAT and NQO1 expression. The jejunal GSH-Px activity showed positive correlations (*P* < 0.05) with plasma CAT, GSH-Px, SOD, and T-AOC activities; colonic T-AOC activity; and jejunal GPx1, GPx4, Nrf1, ATP5A1, ATP5B, and TFAM mRNA levels, while showed negative correlations (*P* < 0.05) with MDA concentration in the plasma and colon. The jejunal T-AOC activity showed positive correlations (*P* < 0.05) with plasma CAT, GSH-Px, SOD, and T-AOC activities; colonic GSH-Px and SOD activities; and jejunal Keap1 and Nrf1 expressions. In the colon, *Bifidobacterium* abundance was positively correlated (*P* < 0.05) with colonic GSH-Px and SOD activities, and GPx4 expression. Besides, colonic *Lactobacillus* abundance was negatively correlated with colonic GSH activity and MDA concentration.

## 4. Discussion

In humans, maternal nutritional strategies during gestation and lactation have been investigated due to their potential impact on fetal growth and development, as well as the beneficial effects on offspring's health [32]. Moreover, maternal adverse nutritional conditions may alter the structure and function of particular organs of offspring and lead to many complications later in life [33]. Previous studies have found that the administration of probiotics and prebiotics during gestation and lactation is a possible dietary strategy to benefit infant health [34]. Probiotics present many beneficial effects, and strain-specific probiotics can exhibit antioxidant activity and reduce the intestinal damage caused by oxidation [35]. The present study investigates whether dietary probiotic or synbiotic supplementation to sows during gestation and lactation affects the antioxidant capacity and mitochondrial function in offspring piglets and further explored whether it is associated with intestinal bacteria. We found that maternal probiotic or synbiotic supplementation during gestation and lactation significantly enhanced systemic and intestinal antioxidant capacity, improved mitochondrial biogenesis, and altered the jejunal and colonic bacteria communities in offspring piglets. Furthermore, correlation analysis revealed that jejunal and colonic microbiota abundances were significantly correlated with antioxidant enzyme activities and mitochondrial biogenesis-related indexes.

The antioxidant capacity in plasma reflects the host's systemic capacity to respond to oxidative damage. H_2_O_2_, as a major type of ROS, is involved in lipoperoxidation [36]. The MDA is a decomposition product of lipoperoxidation, is the important marker of oxidative stress [37]. By lowering the MDA level in plasma, it is possible to lower the degree of lipid destruction and enhance the ability of ROS scavenging. In the present study, dietary probiotic supplementation to sows decreased plasma MDA and H_2_O_2_ concentrations of offspring piglets. The major GSH-dependent enzymatic antioxidants are SOD, GSH-Px, and CAT, which play a vital role in scavenging ROS [38]. The GSH serves as the major endogenous antioxidant, acting as a free radical scavenger in the cell. GSH-px is a key enzyme to catalyze GSH into GSSG. Our results showed that the activity of GSH-Px in the plasma of probiotic- or synbiotic-treated piglets was significantly higher than those in the control group. However, the GSH content in the plasma of probiotic- or synbiotic-treated piglets was increased numerically but not significantly. Based on these results, we speculated that maternal probiotic or synbiotic supplementation may contribute slightly to regulate the oxidation and reduction reactions of GSH; however, the exact mechanism remained unknown. The redox status of GSH can be expressed by its half-cell redox potential (GSH/GSSG Eh) [39]. Further studies are needed to explore the effect of maternal probiotic or synbiotic supplementation on the GSSG and GSH/GSSG Eh levels to fully understand the GSH redox status.

The intestinal mucosa, the front-line barrier to food antigens, pathogens, and commensal organisms, plays a crucial role in sustaining intestinal epithelial homeostasis [40]. However, many stimuli factors (i.e., infection and inflammation) can induce the overproduction of proinflammatory cytokines and ROS to damage intestinal barrier function [41]. In the present study, consistent with the findings in plasma, the MDA content in the colonic mucosa of maternal synbiotic-treated piglets was significantly decreased. Furthermore, maternal probiotic or synbiotic supplementation can partially increase the antioxidant enzyme activities in the jejunum and colon of offspring piglets. Gu et al. reported that isomalto-oligosaccharide and *Bacillus* supplementation to sows during late gestation could improve the placental antioxidant capacity and the piglets' birth weight [42]. In a human study, it was found that plasma and erythrocyte MDA levels were significantly higher, and the erythrocyte GSH level was lower in the pregnant women than in the nonpregnant women, and there was a significant positive correlation in MDA, GSH-R, and GSH-P levels between maternal and cord blood erythrocyte. These results indicated that the fetus may affect by the oxidant status of pregnant women [8]. Synbiotic supplementation could improve the T-AOC level and slightly reduce MDA level in human breastmilk [43]. However, the effect of dietary probiotic or synbiotic supplementation on the antioxidant enzymes in sows' breastmilk or placenta is unclear. Further studies are necessary to explore the effect of probiotic or synbiotic on the transmission of antioxidant capacity from sows to their offspring.

The Nrf2 pathway plays an important role in the regulation of intracellular redox status [44]. We further confirmed that the relative expression of Nrf2 mRNA was increased in the jejunum of the synbiotic group. In addition, dietary synbiotic treatment upregulated the Nrf2-regulated genes, including CuZnSOD (SOD1) and GPx1. These findings indicated that maternal synbiotic supplementation could enhance the antioxidant capacity of offspring piglets by upregulating the Nrf2 pathway. The ATP production and utilization are very active in the intestinal epithelial cells [45], due to the intestinal epithelial cells maintain cell turnover every 3-5 days [46]. Therefore, energy deficits can easily lead to impaired intestinal barrier function. Mitochondria is one of the key sources of oxidative stress, as it utilizes oxygen for cellular ATP production. Oxidative stress in cells can lead to impaired mitochondrial function, including the content of mtDNA and the expression of mitochondrial genes [47]. In the present study, the jejunal ATP concentration in piglets tended to increase after the addition of probiotic or synbiotic in the sows' diets. Furthermore, maternal synbiotic supplementation significantly upregulated the mRNA expression of jejunal ATP5A1 and ATP5B, which might be responsible for the increased ATP level in the synbiotic-treated piglets. An increase in TFAM expression was observed in the jejunal mucosa of maternal synbiotic-treated piglets. It has been reported that TFAM plays a critical role in mitochondrial biogenesis and regulates the mtDNA copy number [48]. Collectively, these findings indicated that maternal probiotic or synbiotic supplementation might improve the processes of jejunum energy metabolism in offspring piglets. Moreover, in the present study, antibiotic and probiotic treatment increased the activity of colonic mitochondrial complex I, which could result in a boost of mitochondrial energy generation. Similar findings were observed in cardiac mitochondrial dysfunction of insulin-resistant rats treated with prebiotics, probiotics, and synbiotics which showed improved cardiac mitochondrial function and reduced oxidative stress [49].

Intestinal microbiota plays a crucial role in maintaining health and regulating pathogenesis in the host. Probiotics can stimulate the colonization of the piglet's gut with beneficial bacteria, enriches the gut microbiota diversity, and prevents the intestinal infection of neonatal piglets [50]. Moreover, probiotics could also exert antioxidant effects by modulating the composition of intestinal microbiota [35]. In humans, supplementing probiotic to the pregnant mother during late pregnancy could promote the colonization of the infant's gut with beneficial bacteria such as *L. rhamnosus GG* or *Bifidobacteria* [51, 52]. Our study in pigs showed that maternal probiotic supplementation significantly increased the *Bacteroidetes* and *Bifidobacterium* abundances in the jejunum and *Bifidobacterium* in the colon of offspring piglets. *Bacteroidetes* are known as short-chain fatty acids producing bacteria, which protects the mocusa of the host from damage by pathogens, provides nutrition for colonic epithelial cells, and reduces inflammation. In addition, *Bacteroidetes* can supply energy to the host via carbohydrate degradation and associated with the immune response of the host [53]. The present study also found that maternal synbiotic supplementation during gestation and lactation increased the relative abundances of Firmicutes, *Bacteroidetes*, *Bifidobacterium*, and *Lactobacillus* in the jejunum of piglets. Firmicutes were found associated with the degradation of different carbon sources, oligosaccharides, proteins, and amino acids and their fermentative metabolisms to provide energy to the host [54]. Furthermore, *Lactobacillus* and *Bifidobacterium*, as the most common probiotic species, could inhibit the growth of pathogenic bacteria to maintain the balance of the intestinal microbiota and thus alleviate the intestinal oxidative stress [55, 56]. Previous studies research also reported that changes in the intestinal microbiota were strongly associated with oxidative stress in high-fat diet-fed mice [57]. Consistent with these findings, the correlation analysis in the present study showed that the antioxidant enzyme activities exhibited a positive correlation with the abundances of Firmicutes, *Bacteroidetes*, *Bifidobacterium*, and *Lactobacillus* while MDA level was negatively correlated with the abundances of jejunal *Bifidobacterium* and colonic *Lactobacillus*. Collectively, these findings indicated that maternal probiotic or synbiotic supplementation alleviated the intestinal oxidative stress in offspring piglets, which might be attributed to the increase in the abundances of beneficial bacteria, such as *Bifidobacterium*.

## 5. Conclusions

**Figure 5:**
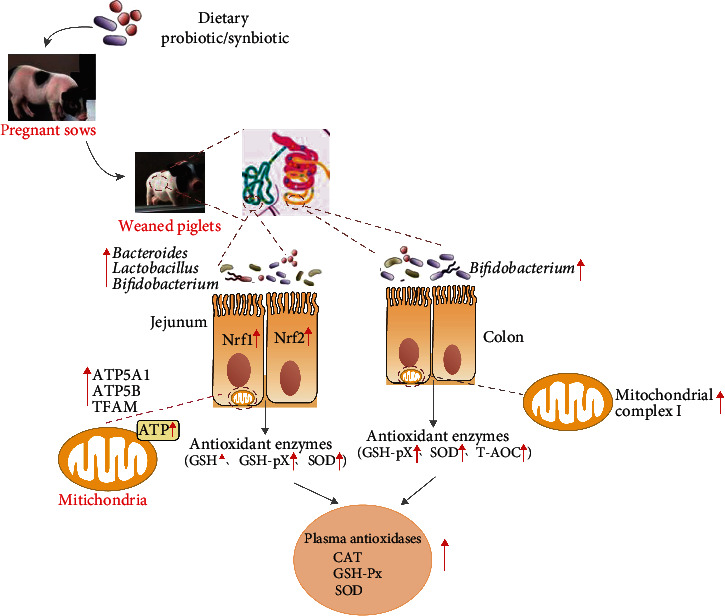
Schematic demonstrated the hypothesized mechanism of maternal probiotic or synbiotic effects on antioxidant capacity of offspring piglets. CAT: catalase; GSH: glutathione; GSH-Px: glutathione peroxidase; SOD: superoxide dismutase; T-AOC: total antioxidant capacity; Nrf1: nuclear factor erythroid 2-related factor 1; Nrf2: nuclear factor erythroid 2-related factor 2; ATP5A1: adenosine triphosphate synthase alpha subunit; ATP5B: adenosine triphosphate synthase *β*, polypeptide; TFAM: mitochondrial transcription factor A.

The present study showed a beneficial effect of maternal probiotic (*Lactobacillus Plantarum* and *Saccharomyces cerevisiae*) or synbiotic (probiotic + xylo-oligosaccharides) supplementation during gestation and lactation on antioxidant capacity and mitochondrial biogenesis, which may partly be attributed to altered intestinal microbiota in offspring weaned piglets. However, maternal synbiotic supplementation did not show any enhanced effects compared to probiotic in offspring weaned piglets (Figure 5). These findings implicated that probiotic or synbiotic would be potential maternal dietary additives to improve offspring's intestinal health.

## Figures and Tables

**Table 1:** Primer sequences used for intestinal mucosa antioxidant-related genes and mitochondrial-related genes.

Genes^a^	Primers (5′-3′)	Size (bp)	Accession NO.
*β*-Actin	F: GATCTGGCACCACACCTTCTACAAC	107	XM_021086047.1
	R: TCATCTTCTCACGGTTGGCTTTGG		
CAT	F: AGCCTACGTCCTGAGTCTCTGC	90	NM_214301.2
	R: TCCATATCCGTTCATGTGCCTGTG		
GPx1	F: TGCTCATTGAGAACGTAGCGT	161	NM_214201.1
	R: CAGGATCTCCCCATTCTTGGC		
GPx4	F: GATTCTGGCCTTCCCTTGC	173	NM_214407.1
	R: TCCCCTTGGGCTGGACTTT		
Keap1	F: CGCCTCATCGAGTTCGCTTACAC	107	NM_001114671.1
	R: GCACGGACCACACTGTCAATCTG		
CuZnSOD	F: CCAGTGCAGGTCCTCACTTCAATC	172	NM_001190422.1
	R: CGGCCAATGATGGAATGGTCTCC		
MnSOD	F: GGACAAATCTGAGCCCTAACG	159	NM_214127.2
	R: CCTTGTTGAAACCGAGCC		
NQO1	F: GTGGAAGCCGCAGACCTTGTG	83	NM_001159613.1
	R: CGTTCAAACCAGCCTTTCAGAATAGC		
Nrf1	F: CGATGCTTCAGAATTGCCAACTACAG	125	XM_021078993.1
	R: GCGTTGTCTGGATGGTCATCTCAC		
Nrf2	F: CCAATTCAGCCAGCACAACACATC	149	XM_003133500
	R: GACTGAGCCTGGTTAGGAGCAATG		
ATP5A1	F: ACGCCATTGATGGAAAGGGT	98	NM_001185142.1
	R: TGGTTCCCGCACAGAGATTC		
ATP5B	F: CATGTTGGGCTTTGTGGGTC	139	XM_001929410.4
	R: ATAGTCTCTGGCAGGCTGGA		
PGC1*α*	F: ATGGAGCAATAAAGCGAAGAGCATTTG	101	NM_213963.2
	R: GAGGAGGGTCATCATTTGTGGTCAG		
TFAM	F: AAATTGCTGAGCTGTGGAGGGAAC	82	NM_001130211.1
	R: TACACCTGCCAGTCTGCCCTATAAG		

^a^CAT: catalase; GPx1: glutathione peroxidase 1; GPx4: glutathione peroxidase 4; Keap1: kelch-like ECH-associated protein 1; CuZnSOD: copper- and zinc-containing superoxide dismutase; MnSOD: manganese-containing superoxide dismutase; NQO1: nicotinamide adenine dinucleotide (phosphate) dependency quinone oxidoreductase 1; Nrf1: nuclear factor erythroid 2-related factor 1; Nrf2: nuclear factor erythroid 2-related factor 2; mtDNA: mitochondrial DNA; ATP5A1: adenosine triphosphate synthase alpha subunit; ATP5B: adenosine triphosphate synthase *β*; PGC-1*α*: peroxisome proliferator-activated receptor *γ* coactivator-1*α*; TFAM: mitochondrial transcription factor A.

**Table 2:** Primer sequences used for intestinal mucosa mtDNA copy number analysis.

Gene^a^	Primers (5′-3′)	Size (bp)	Accession NO.
mt D-loop	F: GATCGTACATAGCACATATCATGTC	198	AF276923
	R: GGTCCTGAAGTAAGAACCAGATG		
G6PC	F: AAGCCAAGCGAAGGTGTGAGC	165	NM_001113445.1
	R: GGAACGGGAACCACTTGCTGAG		

^a^mt D-loop: mitochondria DNA loop; G6PC: glucose-6-phosphatase.

**Table 3:** Primer sequences used for bacteria 16S rRNA.

Bacteria	Sequence 5′-3′	Length (bp)	Reference	Annealing temp, °C
Total bacteria	F: GTGSTGCAYGGYYGTCGTCA	123	[26]	60
	R: ACGTCRTCCMCNCCTTCCTC			
*Bacteroidetes*	F: GGARCATGTGGTTTAATTCGATGAT	126	[27]	60
	R: AGCTGACGACAACCATGCAG			
*Firmicutes*	F: GGAGYATGTGGTTTAATTCGAAGCA	126	[27]	60
	R: AGCTGACGACAACCATGCAC			
*Bifidobacterium*	F: TCGCGTCYGGTGTGAAAG	128	[28]	62
	R: GGTGTTCTTCCCGATATCTACA			
*Clostridium cluster* IV	F: GCACAAGCAGTGGAGT	240	[29]	54
	R: CTTCCTCCGTTTTGTCAA			
*Escherichia coli*	F: CATGCCGCGTGTATGAAGAA	95	[30]	62
	R: CGGGTAACGTCAATGAGCAAA			
*Lactobacillus*	F: AGCAGTAGGGAATCTTCCA	345	[31]	62
	R: ATTCCACCGCTACACATG			

**Table 4:** Effect of maternal probiotic or synbiotic supplementation during gestation and lactation on piglets' antioxidant-related gene expressions in the jejunum and colon.

Items^a^	Dietary treatment				SEM	*P* values
	Control	Antibiotic	Probiotic	Synbiotic		
Jejunum						
CAT	1.12	0.89	1.66	1.61	0.143	0.181
GPx1	1.05	1.38	1.62	1.67	0.093	0.049
GPx4	1.11	1.26	1.49	1.35	0.090	0.482
Keap1	1.02	0.93	1.09	1.12	0.036	0.305
CuZnSOD	1.09^b^	1.01^b^	1.26^ab^	1.82^a^	0.102	0.020
MnSOD	1.05	1.22	1.25	1.52	0.077	0.188
NQO1	1.18	1.49	1.18	1.29	0.142	0.875
Nrf1	1.02^b^	0.95^b^	1.06^ab^	1.31^a^	0.038	0.002
Nrf2	1.11^b^	1.12^b^	1.33^ab^	1.63^a^	0.072	0.032
Colon						
CAT	1.05	0.74	0.82	0.78	0.049	0.076
GPx1	1.03	0.71	0.90	1.00	0.053	0.146
GPx4	1.08^a^	0.38^b^	0.90^ab^	0.95^ab^	0.087	0.020
Keap1	1.00	0.96	1.04	0.94	0.028	0.618
CuZnSOD	0.95	0.57	0.86	0.88	0.059	0.117
MnSOD	1.06	0.95	1.25	1.13	0.070	0.516
NQO1	1.15	0.64	0.87	1.20	0.109	0.269
Nrf1	1.02^a^	0.77^b^	0.93^ab^	1.03^a^	0.033	0.018
Nrf2	1.07^a^	0.64^b^	0.75^ab^	0.73^ab^	0.056	0.014

Data are expressed as means with SEM (*n* = 8). Means with different superscript letters in the same row were significantly different (*P* < 0.05). Values with no letters mean no statistically significant differences among the groups (*P* > 0.05). ^a^CAT: catalase; GPx1: glutathione peroxidase 1; GPx4: glutathione peroxidase 4; Keap1: kelch-like ECH-associated protein 1; CuZnSOD: copper- and zinc-containing superoxide dismutase; MnSOD: manganese-containing superoxide dismutase; NQO1: nicotinamide adenine dinucleotide (phosphate) dependency quinone oxidoreductase 1; Nrf1: nuclear factor erythroid 2-related factor 1; Nrf2: nuclear factor erythroid 2-related factor 2.

**Table 5:** Effect of maternal probiotic or synbiotic supplementation during gestation and lactation on mitochondrial-related gene expression in the jejunum and colon of piglets.

Items^a^	Dietary treatment				SEM	*P* values
	Control	Antibiotic	Probiotic	Synbiotic		
Jejunum						
mtDNA	1.02	0.997	1.08	0.936	0.0381	0.661
ATP5A1	1.02^b^	0.95^b^	1.17^ab^	1.43^a^	0.057	0.014
ATP5B	1.03^b^	1.07^b^	1.20^b^	1.64^a^	0.066	0.001
PGC1*α*	1.04	0.80	1.18	1.14	0.058	0.110
TFAM	1.05^b^	1.21^ab^	1.28^ab^	1.51^a^	0.055	0.016
Colon						
mtDNA	1.06	1.09	0.98	0.80	0.051	0.192
ATP5A1	1.03	0.75	0.89	0.98	0.042	0.100
ATP5B	1.02	0.93	1.08	1.06	0.041	0.629
PGC1*α*	1.11^a^	0.65^ab^	1.16^ab^	0.60^b^	0.076	0.008
TFAM	1.03^a^	0.71^b^	0.89^ab^	0.86^ab^	0.047	0.106

Data are expressed as means with their SEM (*n* = 8). Values with different letters in the same row were significantly different (*P* < 0.05). Values with no letters mean no statistically significant differences among the groups (*P* > 0.05). ^a^mtDNA: mitochondrial DNA; ATP5A1: adenosine triphosphate synthase alpha subunit; ATP5B: adenosine triphosphate synthase *β*, polypeptide; PGC-1*α*: peroxisome proliferator-activated receptor *γ* coactivator-1*α*; TFAM: mitochondrial transcription factor A.

## Data Availability

The data used to support the findings of this study are included in the article and the supplementary information files.

## Supplementary Materials

Supplementary materials Table S1: composition and nutrient levels of the sows' basal diets (air-dry basis; %). Table S2: composition and nutrient levels of the basal diet for weaned piglets. Figure S1: effect of maternal probiotic or synbiotic supplementation during gestation and lactation on ATP concentrations in the jejunum and colon of piglets. Figure S2: effect of maternal probiotic or synbiotic supplementation during gestation and lactation on mitochondrial complex I and III activities in the jejunum and colon of piglets.
