# Supplementary material for: Maternal Probiotic or Synbiotic Supplementation Modulates Jejunal and Colonic Antioxidant Capacity, Mitochondrial Function, and Microbial Abundance in Bama Mini-piglets
Source: Oxid Med Cell Longev. 2021 May 4;2021:6618874. doi: 10.1155/2021/6618874 (PMC8116152; doi:10.1155/2021/6618874)
Supplement: Supplementary materials — Table S1: composition and nutrient levels of the sows' basal diets (air-dry basis; %). Table S2: composition and nutrient levels of the basal diet for weaned piglets. Figure S1: effect of maternal probiotic or synbiotic supplementation during gestation and lactation on ATP concentrations in the jejunum and colon of piglets. Figure S2: effect of maternal probiotic or synbiotic supplementation during gestation and lactation on mitochondrial complex I and III activities in the jejunum and colon of piglets. [file 6618874.f1.docx]

**Supplementary Material**

**Table S1****: Composition and nutrient levels of the sows’ basal diets (air-dry basis; %)**

| **Items** | **Pregnant sows’ diet** | **Lactating sows’ diet** |
| --- | --- | --- |
| Ingredients |  |  |
| Corn | 37.50 | 66.00 |
| Soybean meal | 9.50 | 25.00 |
| Wheat bran | 14.00 | 5.00 |
| Barley | 25.00 |  |
| Soybean hull | 10.00 |  |
| Pregnant sows’ premix^1^ | 4.00 |  |
| Lactating sows’ premix^2^ |  | 4.00 |
| Total | 100.00 | 100.00 |
| Nutrient levels^3^ |  |  |
| Digestible energy (MJ/Kg) | 12.55 | 13.87 |
| Crude protein | 12.82 | 16.30 |
| Crude fiber | 4.56 | 2.87 |
| SID Lys | 0.48 | 0.75 |
| SID Met+ Cys | 0.43 | 0.51 |
| SID Thr | 0.37 | 0.53 |
| SID Trp | 0.13 | 0.17 |
| Calcium | 0.62 | 0.65 |
| Phosphorus | 0.47 | 0.50 |

**Note:** ^1^Pregnant sows’ premix provided the following per kg of diets: CaHPO_4_⋅2H_2_O 10 g, NaCl 4 g, CuSO_4_⋅5H_2_O 80 mg, FeSO_4_ ⋅H_2_O 360 mg, ZnSO_4_⋅H_2_O 240 mg, MnSO_4_⋅H_2_O 100 mg, MgSO_4_⋅7H_2_O 1 g, 1% ICl 50 mg, 1% Na_2_SeO_3_ 36 mg, 1% CoCl₂ 16 mg, NaHCO_3_ 1.4 g, VA 10 000 IU, VD_3_ 1 800 IU, VE 20 mg, VK_3_ 2.4 mg, VB_1_ 1.6 mg, VB_2_ 6 mg, VB_6_ 1.6 mg, VB_12_ 0.024 mg, folic acid 1.2 mg, nicotinamide 20 mg, pantothenic acid 12 mg, biotin 0.12 mg, ferrous glycinate 100 mg, choline chloride 1g, phytase 200 mg, fruity 80 mg, limestone 12 g.

^2^Lactating sows’ premix provided the following per kg of the diet: CaHPO_4_⋅2H_2_O 10 g, NaCl 4 g, CuSO_4_⋅5H_2_O 80 mg, FeSO_4_ ⋅H_2_O 360 mg, ZnSO_4_⋅H_2_O 240 mg, MnSO_4_⋅H_2_O 100 mg, 1% ICl 50 mg, 1% Na_2_SeO_3_ 36 mg, 1% CoCl₂ 16 mg, NaHCO_3_ 1.4 g, VA 10 000 IU, VD_3_ 1 800 IU, VE 20 mg, VK_3_ 2.4 mg, VB_1_ 1.6 mg, VB_2_ 6 mg, VB_6_ 1.6 mg, VB_12_ 0.024 mg, folic acid 1.2 mg, nicotinamide 20 mg, pantothenic acid 12 mg, biotin 0.12 mg, Lysine 1.5 g, ferrous glycinate 100 mg, choline chloride 1g, phytase 200 mg, fruity 80 mg, limestone 12 g.

^3^Calculated nutrient levels using values for feed ingredients from the NRC (2012). SID, standard ileum digestible.

**Table S2: Composition and nutrient levels of the basal diet for weaned piglets**

| **Ingredients** | **Ratio, %** | **Nutrients** | **Levels, % (calculated)^2^** |
| --- | --- | --- | --- |
| Corn | 54.92 | DE, MJ/kg | 13.50 |
| Soybean meal | 22.00 | CP | 16.13 |
| Wheat bran | 10.13 | Ca | 0.44 |
| Rice bran | 8.95 | TP | 0.50 |
| Premix^1)^ | 4.00 | Lys | 1.40 |
| Total | 100.00 | Met + Cys | 0.69 |
|  |  | Thr | 0.78 |

Note: ^1^Premix provided the following per kilogram of diets: enzymic preparation 1.2 g, VA 26 000 IU,VD_3_ 10 000 IU,VE 70 IU,VK_3_ 10 mg,VB_1_ 10 mg,VB_2_ 25 mg,VB_6_ 10 mg,VB_12_ 0.075 mg, biotin 0.4 mg, folic acid 5 mg, nicotinamide 100 mg, pantothenic 50 mg, choline 1 600 mg, flavoring agent 500 mg, edulcorant 300 mg, acidulating agent 5 g, Cu (as CuSO_4_·5H_2_O) 230 mg, Mn (as MnSO_4_·H_2_O) 97 mg, Zn (as ZnSO_4_·H_2_O) 218 mg, Fe (as FeSO_4_·H_2_O) 165 mg, I (as Ca(IO_3_)_2_) 0.3 mg, Se (as Na_2_SeO_3_) 0.3 mg, Co (as CoSO_4_·xH_2_O) 0.4 mg, glucose 2.1 g, antioxidants 0.4 g, antimildew agent 1 g, Ca (as CaHPO_4_ and CaCO_3_) 3.42 g, P (as CaHPO_4_ ) 1.155 g.

^2^Calculated nutrient levels using values for feed ingredients from the NRC (2012).


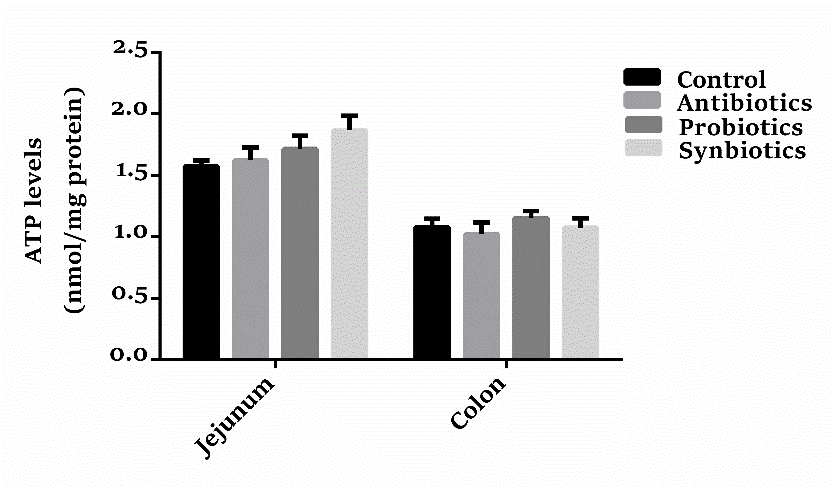


## Supplementary Figure: Effect of maternal probiotics or synbiotics supplementation during gestation and lactation on ATP concentrations in jejunum and colon of piglets. Data are expressed as means ± SEM (*n* = 8). Values with no letters mean none of the differences between the two groups is statistically significant (*P* > 0.05).


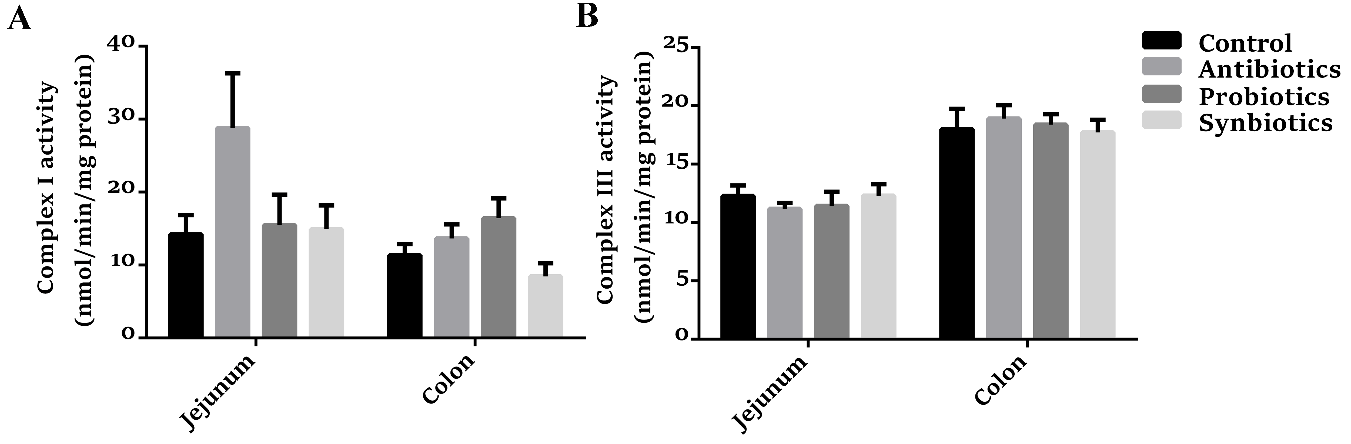


**Supplementary Figure 2:** Effect of maternal probiotics or synbiotics supplementation during gestation and lactation on mitochondrial complex I and III activities in jejunum and colon of piglets. Data are expressed as means ± SEM (n = 8). Values with no letters mean none of the differences between the two groups is statistically significant (*P* > 0.05).
